# Beyond BMI: Nutritional Recovery and Functional Implications of CFTR Modulators in Cystic Fibrosis

**DOI:** 10.3390/biology15040367

**Published:** 2026-02-22

**Authors:** Giovanna Linguiti, Vanja Granberg, Giuseppina Leonetti, Giuseppe Lassandro, Marisa Lassandro, Maurizio Delvecchio, Paola Giordano

**Affiliations:** 1 Pediatric Unit “B. Trambusti”, Cystic Fibrosis Regional Reference Center, Department of Interdisciplinary Medicine, University of Bari “Aldo Moro”, 70124 Bari, Italy; giovanna.linguiti@gmail.com (G.L.); vanjagranberg@libero.it (V.G.); susy.leonetti@virgilio.it (G.L.); giuseppelassandro@live.com (G.L.); marisa.lassandro@gmail.com (M.L.); paola.giordano@uniba.it (P.G.); 2 Department of Biotechnological and Applied Clinical Sciences, University of L’Aquila, 67100 L’Aquila, Italy

**Keywords:** cystic fibrosis, nutrition, body composition, body mass index, ivacaftor, elexacaftor/tezacaftor/ivacaftor, obesity

## Abstract

Cystic fibrosis (CF) is a multisystem genetic disorder in which malnutrition has historically been a major determinant of disease severity. The advent of CFTR modulators has significantly altered the nutritional and functional profile of CF patients. This systematic review, conducted following PRISMA guidelines and registered on PROSPERO, summarizes studies published from 2012 to 2025 that evaluated the impact of CFTR modulators on nutritional status, body composition, and respiratory function, with a particular focus on the relationship between BMI changes and FEV_1_. Seventeen studies including both pediatric and adult populations were identified and analyzed. All studies reported significant increases in BMI following modulator therapy, ranging from +0.9 to +1.6 kg/m^2^ after 6–12 months of elexacaftor/tezacaftor/ivacaftor (ETI), accompanied by improvements in FEV_1_ of 7–13 percentage points. Weight gain was primarily due to increases in fat mass (60–75%). Improvements in albumin, prealbumin, and vitamin levels, along with reductions in C-reactive protein (CRP) and fecal calprotectin, reflected systemic metabolic recovery. However, a marked increase in overweight and obesity (up to 40%), together with increased visceral adiposity, has also been observed. Overall, CFTR modulators result in significant nutritional and functional improvements, highlighting the need for strategies that prioritize overall metabolic health rather than weight gain alone.

## Simple Summary

Cystic fibrosis is a genetic disease that affects multiple organs and often leads to poor growth and nutrition. Recently, new medicines called CFTR modulators have changed the way the disease affects patients. This review looked at studies published between 2012 and 2025 to understand how these treatments influence body weight, body composition, and lung function. We found 17 studies involving children and adults. Most patients gained weight after starting these medicines, mainly due to an increase in fat rather than muscle. Lung function also improved, showing that the body worked better overall. Blood tests and other markers suggested that metabolism and nutrition improved with treatment. However, many patients became overweight or obese, and fat around organs increased. This shows that while CFTR modulators help people grow and breathe better, it is important to focus on gaining healthy body composition rather than just weight. Understanding these changes can help doctors provide better care and guide patients toward healthier outcomes.

## 1. Introduction

Cystic fibrosis (CF) is an autosomal recessive genetic disorder caused by mutations in the *CFTR* (Cystic Fibrosis Transmembrane Conductance Regulator) (OMIM: 602421) gene, which result in defective regulation of chloride and bicarbonate transport across epithelial tissues. This dysfunction results in thick, viscous mucus secretions, predisposing individuals to chronic respiratory infections, exocrine pancreatic insufficiency, and intestinal malabsorption [1].

In addition to respiratory complications, malnutrition has long been one of the key clinical features of the disease. Pancreatic insufficiency, nutrient malabsorption, increased energy expenditure, and chronic inflammatory status contribute to negative energy balance, resulting in low body weight and reduced body mass index (BMI).

Studies conducted in the pre-modulator era, such as that by Gramegna et al. (2022) [2], have documented a strong positive correlation between BMI and FEV_1_. An adequate BMI is associated with better respiratory function (FEV_1_), a lower risk of infections, and longer survival, whereas malnutrition and low BMI are independent indicators of pulmonary decline and mortality [2,3]. While BMI gain remains a prognostic indicator, the emergence of “normal weight obesity” and visceral fat accumulation may introduce new metabolic risk that could eventually blunt these functional gains.

The advent of CFTR modulators, starting with ivacaftor for gating mutations and progressing to the triple combination elexacaftor/tezacaftor/ivacaftor (ETI), has revolutionized the clinical management and natural history of the disease by directly targeting the underlying molecular defect [4]. These drugs have led to marked improvements in pulmonary function, a reduction in respiratory exacerbations, and a significant increase in BMI, marking a transition from a catabolic, low nutritional status phenotype to a more favorable metabolic balance.

At the same time, a progressive alignment of anthropometric parameters in patients with CF to those of the healthy population has been observed, indicating improved nutritional recovery and enhanced metabolic efficiency [5]. However, this positive evolution is accompanied by the emergence of new clinical challenges, such as an increased risk of overweight and obesity, changes in body composition (an increase in fat mass relative to lean mass), and potential metabolic alterations.

In this context, it becomes essential to reassess the relationship between nutrition and respiratory function in the era of CFTR modulators. While an increase in BMI continues to represent an indicator of better prognosis, it remains unclear whether weight gain reflects a true recovery of functional lean mass or rather an accumulation of adipose tissue with long-term metabolic implications.

The present review aims to systematically examine the current evidence on the impact of CFTR modulators on nutritional status, body composition, and respiratory function in patients with CF, with a particular focus on the relationship between changes in BMI and FEV_1_ as integrated indicators of clinical improvement.

## 2. Materials and Methods

### 2.1. Study Selection

A systematic literature search was conducted in the PubMed and Web of Science databases, limited to the period from January 2012 to September 2025. Keywords were combined using the Boolean operators “AND” and “OR” were: “cystic fibrosis” AND “nutrition” AND “modulators.” The selection process followed PRISMA guidelines and all references correspond to peer-reviewed articles published online-first or early-access. No preprints or non-peer-reviewed data were included to maintain high methodological rigor.

**Figure 1:**
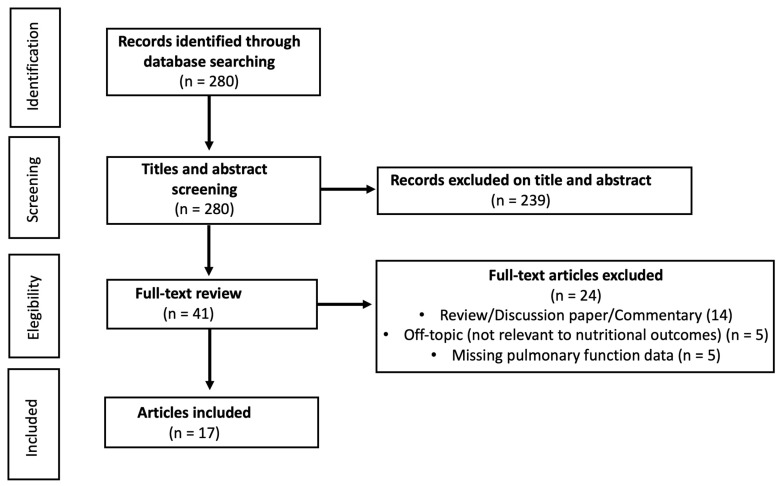
Flow chart of study inclusion in systematic review.

The search was restricted to English language studies, with no age restrictions, and included both pediatric and adult populations. Initially, 280 articles were identified. Following screening of titles and abstracts, 41 articles were selected for full-text review. Of these, 14 reviews, 5 off-topic articles (not relevant to nutritional outcomes), and 5 articles lacking pulmonary function data (FEV_1_) were excluded. After applying the inclusion and exclusion criteria, 17 studies were included in the final analysis (Figure 1). The protocol was registered in the International Prospective Register of Systematic Reviews (PROSPERO, https://www.crd.york.ac.uk/PROSPERO ID: CRD420251175851, accessed on 24 November 2025).

### 2.2. Eligibility Criteria

The selection of articles was conducted according to the PICOS framework (Population, Intervention, Comparison, Outcomes, Study design) (Table 1).

The inclusion criteria were as follows: (i) confirmed diagnosis of CF; (ii) treatment with CFTR modulators; (iii) availability of nutritional data, expressed as changes in weight, BMI, BMI z-score, or body composition parameters (fat-free mass (FFM), fat mass (FM), body cell mass (BCM)); (iv) availability of respiratory function data, specifically FEV_1_ or ppFEV_1_ (pre- and post-treatment or comparison with controls); (v) studies conducted in humans, published in full text between 2012 and 2025; (vi) observational, prospective, retrospective study designs, or clinical trials; (vii) review articles were excluded, but their reference lists were screened for potentially eligible studies.

The exclusion criteria were: (i) animal or in vitro studies; (ii) studies reporting only baseline data without follow-up; (iii) abstracts, editorials, letters, or conference papers lacking complete data; (iv) studies that did not report nutritional or respiratory parameters.

### 2.3. Data Extraction and Synthesis

Two independent reviewers (V.G. and G.Lin.) conducted study selection and data extraction, resolving any discrepancies by consensus. For each included study, the following data were extracted: (i) author, year, and study design; (ii) type of modulator and duration of follow-up; (iii) number of patients; (iv) main nutritional and functional outcomes (Table 2).

The results were organized and discussed in three main sections: Nutritional status trend after initiation of CFTR modulators; Body composition: Prevalence of Fat Mass Gain and Adipose tissue distribution; Nutritional status, metabolism and respiratory function.

### 2.4. Risk of Bias Assessment

The methodological quality of the observational studies included in this review was assessed using an adapted version of the Newcastle–Ottawa Scale (NOS), which evaluates three main domains: (i) Selection of participants (0–4 points); (ii) Comparability of groups (0–2 points); (iii) Outcome assessment (0–3 points).

The NOS assessment was conducted independently by two reviewers, with any discrepancies resolved by consensus. According to the adapted scale, most studies were of high methodological quality (*n* = 14; 82.4%), while the remaining studies were classified as moderate quality (*n* = 3; 17.6%). No study was rated as low quality.

High-quality studies were characterized by adequate participant selection, clear definitions of inclusion and exclusion criteria, good group comparability, and objective, standardized measurement of outcomes (BMI, FEV_1_, biochemical parameters). Moderate-quality studies, on the other hand, exhibited limitations in group comparability, follow-up duration, or the use of self-reported data for certain nutritional variables. Furthermore, small sample sizes in several included studies may limit the generalizability of the findings and reduce statistical power (Table S1).

## 3. Results

### 3.1. Nutritional Status Trends After Initiation of CFTR

In different studies analyzed, the initiation of therapy with CFTR modulators—in particular the triple combination elexacaftor/tezacaftor/ivacaftor (ETI)—is consistently associated with increases in body weight and BMI, both in children and adults.

In adults treated with ETI, observational cohort studies of medium to large series show a mean increase in BMI of about +1 to 1.5 kg/m^2^ in the first year, with the greatest increase typically occurring within the first 3–6 months and then a plateau. Petersen et al. [9], in a cohort of 134 adults, describe an increase in BMI growth rate of +1.47 kg/m^2^/year after the start of ETI, compared to the previous period, with a mean follow-up of 12 months. Similarly, Taelman et al. [10] observed an average increase of +1.2 kg/m^2^ at 18 months in 17 adults, with most of the gain concentrated in the first 3 months and then maintained over time.

The multicenter study by Solís-García et al. [14] (108 adults) confirms this pattern: the average BMI goes from 21.9 to 23.0 kg/m^2^ after one year of ETI, with a reduction in the share of underweight (from 9.3% to 1.9%) and a significant increase in overweight/obese subjects (from 8.3% to 22.9%).

Other works document comparable weight gain: Hevilla et al. [13] report an average gain of ~5 kg after one year of ETI in 31 adults, with a net reduction in the prevalence of malnutrition and an increase in cases of overweight/obesity; Caley et al. [11], in 40 adults, show an increase in BMI from 23.0 to 24.6 kg/m^2^ in about 5 to 6 months of ETI, with a final share of overweight/obesity of 41% of the cohort. Interestingly, in this study, weight gain is not necessarily accompanied by increased energy intake. In this specific case, the caloric intake decreases from 2551 to 2153 kcal/day, while the BMI increases significantly.

Loel et al. [19], in 40 Australian adults, observe something very similar: the energy intake drops from 139.3 to 116.6 kJ/kg/day after about 9 months of ETI, but the BMI still increases significantly; at the same time, there is a slight better “quality” of the diet (more vegetables, fewer “discretionary” foods), even with saturated fats still high.

On the metabolic and micronutritional side, several studies converge on better absorption and normalization of some markers. Patel et al. [17] (136 adolescents/adults) document, after ETI, an increase in BMI and a significant increase in total cholesterol, LDL, HDL and non-HDL, with values on average still in the normal range; plasma vitamins A and D also increase, while vitamin E remains stable. There is no linear correlation between the magnitude of the ΔBMI and the increase in lipids or vitamins. Schembri et al. [5] in a pediatric cohort (54 children 5–15 years) observed a modest but significant increase in vitamin A after ETI and reported a small share (6%) of patients with serum values above the reference range; vitamin D and E showed no significant changes. Hevilla et al. [13] report, in addition to weight gain, an improvement in vitamin profiles and markers of absorption, suggesting a recovery of intestinal and pancreatic function.

Furthermore, data about pediatric patients treated with modulators showed improvement in nutritional status. In particular, Imrei et al. [8] (49 children, 24 months of LUM/IVA) report an increase in BMI z-score from −0.81 to −0.39, with stability of muscle mass and significant increase in fat mass; at the same time, fecal elastase levels improve, especially in younger patients. On the other hand, Tindall et al. [7], in 15 children aged 4–24 months with gating mutations treated with ivacaftor for 12 weeks, observed a significant increase in the weight-for-age z-score and improvements in markers of absorption and pancreatic function (e.g., fecal elastase), with an increase in energy intake and protein share.

In adulthood, studies on ivacaftor confirm a similar picture, albeit to a lesser extent than with ETI. In fact, King et al. [6], in 20 adults with the G551D mutation, describe an overall increase of +2.5 kg in 6 months, with an increase in BMI and especially in fat mass; gains stabilize in the 2-year follow-up. Similarly, the combined analysis of phase III trials by Borowitz et al. [3] shows significant improvements in weight and BMI in patients ≥6 years old with G551D (in adults, approximately +0.9 kg/m^2^ BMI at 48 weeks compared to placebo).

Overall, these data converge on one key point: the introduction of CFTR modulators almost always leads to an improvement in nutritional status, even in the absence of additional overeating, with a reduction in malnutrition but the appearance of an increasing share of overweight and obesity.

### 3.2. Body Composition: Prevalence of Fat Mass Gain and Adipose Tissue Distribution

Studies that directly assess body composition (BIA, DEXA, and TAC) consistently show that post-modulatory weight gain is largely due to the increase in fat mass (FM), rather than lean mass (FFM).

Nutritional status is closely linked to pulmonary function and survival in cystic fibrosis (CF), yet body mass index (BMI) alone is an insensitive marker. BMI does not distinguish fat-free mass (FFM) from fat mass (FM), and individuals with preserved or normal BMI may show marked FFM depletion, reduced bone mineral density, and worse lung function on detailed body composition assessment [21,22,23,24,25]. In both children and adults with CF, FFM derived from dual-energy X-ray absorptiometry (DXA) or bioelectrical impedance analysis (BIA) correlates more strongly with forced expiratory volume in 1 s, respiratory muscle strength, exercise capacity, and markers of disease severity than BMI, whereas FM is often unrelated or inversely associated with pulmonary outcomes [12,21,23,26,27]. Moreover, BMI fails to detect “hidden” FFM depletion and stunting in a substantial proportion of patients [25,28]. Therefore, routine use of DXA or BIA to quantify body composition is recommended to complement BMI and refine nutritional risk stratification in CF [12,21,22,23,26].

Westhölter et al. [15], using a fully automated chest CT segmentation in 66 adults, show that after ETI that all adipose compartments (total, subcutaneous, visceral, epicardial, and intramuscular adipose tissue) increase markedly, with an increase in the total adipose tissue ratio of +46%. The increase in muscle ratio is much more modest (+1.63%), although statistically significant. Initially underweight subjects show the most marked increase in adipose tissue, while muscle gain is relatively uniform across BMI classes.

Navas-Moreno et al. [16], again with chest CT scan and open-source software analysis in 26 adults, confirm this picture: after ETI, an increase in total body volume and, in particular, in subcutaneous, visceral and intermuscular fat is observed; the only muscle compartment that increases significantly is the very low density muscle (VLDMA), indicative of adipose infiltration. In addition, a high proportion of pre-treatment VLDMA is associated with lower final FEV_1_ and less improvement in FEV_1_, identifying this compartment as a potential risk marker.

Studies based on BIA reinforce the concept of predominantly adipose gain. Hevilla et al. [13] report that the average gain of about 5 kg after one year of ETI is composed of 60% FM and 40% FFM, with a reduction in malnutrition but an increase in cases of overweight/obesity. Knott-Torcal et al. [12] (36 adults, 6 months of ETI) document a significant increase in BMI, FM, and visceral fat area, while lean mass parameters do not change substantially. Importantly, %FEV_1_ correlates negatively with FM and visceral fat and positively with body cell mass, suggesting that excess FM—particularly visceral—may be associated with worse respiratory performance, while the active cellular component of lean mass remains protective.

In the pediatric population, Imrei et al. [8] show that, after 24 months of LUM/IVA, the BMI z-score increases while muscle mass remains stable and fat mass increases significantly, highlighting a trend towards a phenotype of “normal-weight obesity”: BMI in the normal range, but unfavorable body composition.

On the muscle function front, the cross-sectional study by Clayton et al. [20] compares 15 people with CF on stable therapy with ETI (with good respiratory function) and 15 age- and sex-matched healthy controls: there are no significant differences in lean mass, fat mass, FFM, nor in peripheral muscle strength and endurance, nor in bone mineral density. In this relatively healthy and active subgroup, therefore, triple therapy appears to be able to “normalize” muscle mass and function compared to the general population.

Overall, body composition data indicate that (i) FM increases much more markedly than FFM; (ii) visceral and intermuscular fat increase; (iii) muscle quality (density) may deteriorate in some patients (increased VLDMA), with possible repercussions on respiratory function; (iv) ETI can restore lean mass and muscle strength in a range comparable to healthy controls in subjects who are already well nourished or with good respiratory function.

### 3.3. Nutritional Status, Metabolism and Respiratory Function

All studies that report nutritional and respiratory parameters simultaneously show that weight improvement is accompanied, at least initially, by an improvement in lung function.

Solís-García et al. [14] documented, after one year of ETI, an increase in BMI from 21.9 to 23.0 kg/m^2^ and an improvement in ppFEV_1_ from 63.9% to 76.8%, with a drastic reduction in exacerbations (patients without exacerbations went from 10.2% to 46.2%; those with >4 exacerbations from 40.7% to 1.9%). Patients who gain more BMI are those with lower BMI and FEV_1_ and greater number of exacerbations at the beginning, suggesting compensatory anabolic recovery in the most compromised subjects.

Westhölter et al. [15] report a median ΔBMI of +1.4 kg/m^2^ and a ΔppFEV_1_ of +10.5 percentage points after ETI, in line with data from pivotal trials and other real-life studies.

These findings confirm that, although nutritional recovery and weight gain represent key therapeutic targets, ETI is capable of producing substantial and clinically significant enhancements in lung function, particularly within the first year of therapy. Such improvements surpass the commonly accepted minimal clinically important difference (MCID) for ppFEV1 and underscore the profound functional impact of highly effective CFTR modulators on respiratory outcomes.

Taelman et al. [10] confirm a favorable combination of nutrition and glucose metabolism: the increase in BMI (+1.2 kg/m^2^ in 18 months) is associated, in a subgroup of 10 patients with CFRD/IGT, with an improvement in glycemic profiles with insulin withdrawal in 3 out of 6 insulin-treated patients and transient normalization of OGTT in some subjects.

Petersen et al. [9] describe, in addition to the improvement in BMI, a reduction in random blood glucose and HbA1c in patients without CFRD, while in subjects with CFRD an increase in total cholesterol, HDL and LDL is observed, confirming a rearrangement of lipid metabolism with possible emergence of cardiovascular risk.

Navas-Moreno et al. [1] add a key piece of information: patients with higher low-density muscle share (VLDMA) before ETI have a lower final FEV_1_ and lower ΔFEV_1_ after therapy, suggesting that not only weight, but muscle quality and fat distribution influence functional response.

Overall, the studies document (i) a parallel improvement in BMI and FEV_1_ in the majority of patients; (ii) a more marked respiratory benefit in subjects with worse nutritional and functional status at baseline; (iii) the possibility that excess FM, particularly visceral and intramuscular, and low muscle quality attenuate functional gain over time; (iv) a favorable impact on glucose metabolism in many patients with CFRD, but with an increase in plasma lipids and blood pressure in a non-negligible share of subjects, with potential long-term metabolic risk.

## 4. Discussion

The set of data from the included studies outlines a true paradigm shift in the natural history of cystic fibrosis. If in the pre-modulator era the dominant problem was caloric-protein malnutrition, with low BMI, lean mass depletion and strong correlation between underweight, worse respiratory function and mortality, today CFTR modulators—in particular ETI—have transformed this scenario, driving a progressive shift toward normal-weight and overweight phenotypes, with a consistent increase in fat mass and an emerging risk of “overnutrition” and metabolic complications.

Studies on real-life ETI [9,10,11,13,14] show a concordant increase in BMI of about 1–1.5 kg/m^2^ in the first year, with a marked reduction in malnutrition but an increase in the share of overweight/obese patients. In Solís-García [14], the prevalence of OW/GO triples in one year, from 8.3% to 22.9%; in Caley’s British multicenter study [11], after ETI, 41% of the adult cohort is overweight/obese.

This trend is consistent with the pediatric data from Imrei [8] and with the ivacaftor cohorts [3,6,7], which already showed weight and growth recovery after correction of the CFTR defect.

Overall, the available evidence suggests that modulator-induced anabolic recovery, with larger increases in BMI and body mass, is more pronounced in patients with more severe disease at baseline (low BMI, reduced FEV_1_, and frequent exacerbations), as highlighted by Solís-García [14].

However, once the BMI threshold considered “optimal” for HR has been exceeded, further weight gain, especially in FM, does not necessarily translate into functional benefit and can even introduce new problems. In this context, BMI increases in the range of overweight or obesity do not affect pulmonary function, highlighting that not all weight gain is beneficial and reinforcing the need to monitor body composition rather than BMI alone [29,30].

Several factors may influence the clinical outcomes of CFTR modulator therapy. Genotype is a major determinant, as the efficacy of modulators varies according to the type of CFTR mutation (e.g., gating, trafficking, or minimal function mutations) [12,31]. In addition to CFTR variants, modifier genes such as *SLC26A9* and others have been shown to contribute to interindividual variability in response, affecting lung function, pancreatic status, and potentially glycemic control [14,32]. Age and disease stage at treatment initiation also play a role, with earlier intervention generally associated with more favorable outcomes, while patients with advanced disease may experience more limited improvements [31,33]. Finally, geographic and population differences can influence observed outcomes in real-world studies. These include variations in access to CFTR modulators due to differences in healthcare systems or availability, differences in the prevalence of specific CFTR mutations across populations, heterogeneity in registry methodologies and follow-up protocols, as well as socio-economic and environmental factors that may affect disease progression and treatment response [33].

A further element that emerged from the comparative analysis of the different studies concerns the marked individual heterogeneity of the nutritional and metabolic response, modulated by factors such as age, type of mutation and duration of exposure to CFTR modulators. In particular, age is a relevant determinant: younger patients show a faster and more pronounced anabolic recovery [9,10,13,14], with more conspicuous increases in BMI and fat mass than adults, probably reflecting greater metabolic plasticity and a more evident role of “catch-up growth”. However, it is precisely in younger subjects—both in ETI therapy and, previously, with ivacaftor or LUM/IVA—that weight gain tends to focus on fat mass.

The CFTR genotype also influences the therapeutic response. In patients with gating mutations treated with ivacaftor [3,6,7], nutritional improvement is constant but more gradual, while in subjects with F508del mutations (homozygous or heterozygous with MF allele) the introduction of ETI results in the most marked weight gain, with more pronounced effects on both body composition and metabolic parameters [9,11,13,14,15]. This phenomenon suggests that the greater functional efficacy of ETI also involves deeper energy rebalancing, but at the price of a higher risk of fat accumulation than other less potent modulators.

The timing of nutritional and functional improvement appears surprisingly rapid [10,11,13,15,19]. Numerous studies indicate that significant gains in weight, BMI, and FM appear in the first few weeks, with a peak improvement within 3–6 months, while a plateau is often observed beyond the first year [10,14]. The same temporal pattern applies to respiratory function, with more marked increases in FEV_1_ in the first months and then stabilization.

The increase in post-ETI BMI is not determined by an increase in caloric intake. The energy input decreases significantly after the start of therapy, while the BMI continues to increase.

These results, in agreement with previous pathophysiological studies of ivacaftor, suggest that weight gain predominantly results from an interplay of different factors which are (i) reduction in energy expenditure, due to less chronic inflammation, better respiratory function, reduction in the work of breathing; (ii) improvement of absorption and digestion due to partial recovery of pancreatic function, normalization of bile flows, reduction in steatorrhea; (iii) normalization of basal and mitochondrial metabolic rate; and (iv) possible remodulation of the appetite–satiety axis and the intestinal microbiota.

In this context, dietary intake often remains quantitatively high and qualitatively suboptimal: in Loel [19], the intake of saturated fats and “discretionary” foods (snacks, sweets, and junk food) remains above recommended levels, despite a slight increase in the vegetable quota.

These findings suggest that the challenge is no longer “reaching enough calories,” but could be managing the excess calories and the quality of macronutrients in an organism that, thanks to modulators, has recovered metabolic efficiency. Studies with TAC [15,16] and BIA/DEXA [8,12,13] show with great consistency that post-modulator weight gain is unbalanced towards fat mass, with marked increase in total and visceral adipose tissue, increase in intermuscular fat, modest or no FFM gain in the majority of adult cohorts, and development of normal-weight obesity patterns in children (BMI in normal age but FM elevated).

In addition, Navas-Moreno [16] shows that it is not only the amount of muscle that counts, but also its density: a higher share of VLDMA (fat-infiltrated muscle) is associated with lower FEV_1_ and less respiratory improvement after ETI.

These data impose a conceptual step: BMI alone is no longer a sufficient indicator in the ETI era, so it is necessary to monitor body composition (FM/FFM), adipose tissue distribution (visceral vs. subcutaneous), muscle quality (density). Functional lean mass (i.e., body cell mass, indexed FFM) emerges as a parameter more closely related to FEV_1_ than simple BMI, as suggested by Knott-Torcal [12] and other works [22,34].

In other words, not all weight gain is “good”: the goal should move from “as much weight as possible” to an optimal body composition, with adequate FFM and control of visceral adiposity.

Historically, pre-modulatory studies had already shown that better nutritional status is associated with better respiratory function and survival. Data in the ETI era confirm and expand this concept: studies by Solís-García, Westhölter, Taelman [7,14,15] and others [35] document a parallel improvement in BMI and ppFEV_1_, with an increase of 8–12 percentage points in FEV_1_ in many cases; the benefit is particularly evident in patients with lower BMI and FEV_1_ and more exacerbations at the beginning of therapy, suggesting that correction of nutritional deficiency and reduction in catabolic status directly contribute to functional recovery.

However, the same data indicate that beyond a certain threshold of BMI—and especially in the presence of high visceral FM, adipose muscle infiltration and low increase in FFM—the functional benefit tends to stabilize or attenuate, and in some cases a long-term decline may emerge, as glimpsed in the pediatric study of Imrei [8] with LUM/IVA (initial respiratory improvement followed by reduction in ppFEV_1_ over time, against an increase in FM).

From a broader biological perspective, the non-linear relationship observed between BMI gain and FEV1 improvement may be interpreted as being consistent with a conceptual energy trade-off [36]. Evidence from other animal systems indicates that increased energy availability does not necessarily result in proportional improvements across all functional traits, as energy may be redistributed in a non-linear manner among storage, immune-related processes, and performance. It should be emphasized that this interpretation remains speculative and is based on conceptual and theoretical analogies rather than on direct clinical evidence in cystic fibrosis. For instance, cross-species studies demonstrate that increased resource allocation to one specific function can diminish performance in another [37]. In the context of CF, although CFTR modulators correct the underlying molecular defect and improve metabolic efficiency, the associated weight gain may reach a threshold at which the accumulation of visceral or deep muscle adipose tissue begins to counterbalance respiratory benefits, reflecting a comparable biological challenge in resource allocation [38].

In summary, the relationship between nutrition and FEV_1_ remains positive, but becomes more complex because the increase in BMI is beneficial as long as it reflects true anabolic recovery (FFM, metabolic normalization), and beyond a certain point, the accumulation of visceral FM and the loss of muscle quality can counterbalance, at least in part, the respiratory advantage.

With the increase in BMI and FM, studies report several signs of metabolic risk such as increase in total, LDL and HDL cholesterol after ETI, especially in patients with CFRD [9,17] and increased mean arterial pressure in some adult cohorts [19], in continuity with what has already been observed for lumacaftor/ivacaftor.

At the same time, several studies [4] show a favorable impact on glucose metabolism (better glycemic control, insulin withdrawal in a part of CFRD patients), but examples of late worsening or new appearance of glycemic dysfunction in subjects who have gained a lot of weight suggest that excess weight may itself become a risk factor for insulin resistance and diabetes [39].

Collectively, these findings indicate that traditional CF nutritional strategies—based on indiscriminate high-calorie and hyperlipid diets—are no longer appropriate for many patients on highly effective modulator therapy.

Hence, there is a need for a new model of nutritional management, which should take into account different factors to personalize treatments: BMI, body composition, age, level of physical activity and presence of metabolic comorbidities, quality food (not just quantity). Furthermore, we suggest structured monitoring of FM, FFM, visceral fat (BIA, DEXA, TAC where available), lipid profile, blood glucose, HbA1c, liver markers, blood pressure, fat-soluble vitamins with possible reduction in supplementation. Diet therapy in CF must evolve from “overeating so as not to lose weight” to “optimize body composition and prevent metabolic risk”.

## 5. Conclusions

CFTR modulators demand a new approach to nutritional management in cystic fibrosis. The goal can no longer be weight gain in isolation, but the maintenance of a favorable and metabolically efficient body composition, in which fat-free mass and muscle quality play a central role.

Nutritional assessment should therefore evolve toward more comprehensive parameters (such as muscle density, adipose tissue distribution, and metabolic profile) surpassing the simple monitoring of BMI. Only a personalized approach, capable of balancing nutritional recovery with the prevention of metabolic risk, can ensure that the systemic benefits of CFTR modulators translate into genuine improvements in respiratory health and long-term prognosis. However, given the high inter-individual variability in response to these treatments, all clinical interventions must be carefully tailored to the patient’s specific metabolic profile and closely monitored by the multidisciplinary team to ensure safety and efficacy.

Finally, we draw attention to the clinical relevance of normal-weight obesity and muscle density, highlighting the potential risk of overweight and obesity in these patients as an emerging priority for pediatricians and dietitians in cystic fibrosis management.

To address these challenges, clinicians should move beyond BMI by integrating body composition analysis (e.g., BIA or DXA) into routine follow-ups to monitor lean mass quality. It is crucial to implement standardized metabolic screenings and transition nutritional counseling toward strategies that promote metabolic stability, ensuring that long-term care balances pulmonary gains with systemic health.

## Figures and Tables

**Table 1:** PICOS model.

Population	Pediatric and Adult Patients with a Confirmed Diagnosis of Cystic Fibrosis
Intervention	Treatment with CFTR modulators (ivacaftor, lumacaftor/ivacaftor, tezacaftor/ivacaftor, elexacaftor/tezacaftor/ivacaftor).
Comparison	Pre-treatment conditions or control populations not receiving modulators, where available.
Outcomes	Changes in nutritional status (weight, BMI, body composition) and respiratory function (FEV_1_ or ppFEV_1_).
Study design	Prospective, retrospective, or cohort studies.

**Table 2:** Summary of cited studies.

Author and Year	Study Design	N	Age Range	Follow-Up Duration	CFTR Modulators	ΔBMI	Baseline FEV_1_ (% Predicted)	Major Findings
Borowitz et al. (2016) [3]	Post hoc analysis of two international, multicenter, randomized, double-blind, placebo-controlled phase 3 trials	213	12 ± 4.2 30 ± 8.2	48 weeks	Ivacaftor	+0.9 kg/m^2^ (Patients > 20 years) +0.26 (BMI-for-age z-score, patients < 20 years)	60% (patients > 20 years) 77.7% (patients < 20 years)	Nutritional status improved following treatment with ivacaftor for 48 weeks
King et al. (2021) [6]	Single-center, double-blind, placebo-controlled, randomized, crossover trial	20	32.5 years (18–65), median	28 days on ivacaftor and 28 days on placebo (crossover) 5 months open-label ivacaftor 2.5 years total ivacaftor exposure for 11 participants	Ivacaftor	+0.8 kg/m^2^ (after 6 months) +1.2 kg/m^2^ (after 2.5 years)	54.5% (range 23–99%)	Small gains were seen in FFM in the first month of ivacaftor treatment. Weight, BMI, and fat-mass gains in the first 6 months on ivacaftor plateaued by 2.5 y. The metabolic and clinical consequences of weight and FM gains remain to be determined
Tindall et al. (2023) [7]	Prospective longitudinal multicenter study	15	Children 4–24 months	12 weeks	Ivacaftor	+0.36 (BMI-for-age z-score, 12 weeks)	N/A	Overall, younger children experienced favorable changes in nutritional and growth status in the first 12 weeks of ivacaftor therapy
Imrei et al. (2025) [8]	Real-world observational study	49; 42 completed 24-month follow-up	9.3 years (range 5.5–14.2), median	24 months	Lumacaftor/Ivacaftor (LUM/IVA)—dual therapy	+0.42 (BMI z-score, Overall)	102% (Overall median)	The increase in BMI was primarily driven by gains in FM rather than muscle mass. Lung function (FEV_1_) showed an initial improvement during the first year of therapy, followed by a subsequent decline. These findings suggest a metabolic shift toward greater adipose deposition, which may predispose patients to NOW, a phenotype associated with poorer FEV_1_ outcomes
Petersen et al. (2022) [9]	Single-center, retrospective, observational study	134	33.6 ± 10.6	12.2 months	ETI	+1.47 kg/m^2^	57.9% (±24.1)	In this single-center, retrospective, observational study of 134 adults with CF, initiation of elexacaftor-tezacaftor-ivacaftor was associated with increases in BMI at a mean follow up of 12.2 months. Changes in other cardiometabolic risk factors were also observed. Widespread use of elexacaftor-tezacaftor-ivacaftor may be expected to increase the incidence of overnutrition in the CF population
Taelman et al. (2023) [10]	Retrospective, observational, single-center cohort study	17	31.3 ± 9.0	BMI: 18 months before baseline and 18 months after therapy initiation Glycemic outcomes (subgroup): up to 36 months after ETI initiation	ETI	+1.2 kg/m^2^	66.0% (±24.3)	After initiation of ETI an increase in BMI was observed in adults with CF. ETI can have a beneficial impact on glucose metabolism in patients with CFRD, leading to a possible need for reduction or cessation of insulin therapy
Caley et al. (2023) [11]	Multicenter Prospective Observational Study	Pre–post ETI group: *n* = 40 with CF who started ETI between study time points Pre-ETI comparison group: *n =* 10 with CF not on any modulator therapy	≈35.6 years (estimated from grouped age data)	Time between baseline and follow-up: median 68 weeks (range 20–94) Duration on ETI at follow-up: median 23 weeks (range 7–72)	ETI	+1.6 kg/m^2^ (after 12 months)	49.4% (±19.4)	These findings tentatively suggest that the increase in BMI with ETI therapy may not simply be attributable to an increase in oral intake. Further exploration into the underlying etiology of weight gain with ETI therapy is needed
Schembri et al. (2023) [5]	Retrospective bservational study	54	11.5 years (range 5–15), median	Median 171 days after starting ETI	ETI	+0.35 (BMI z-score)	83.0%	ETI therapy significantly improves BMI z-score and vitamin A and E levels in children. Blood levels must be monitored to prevent hypervitaminosis A following treatment initiation
Knott-Torcal et al. (2023) [12]	Prospective observational study	36	30.4 ± 8.7	6 months	ETI	+1.2 kg/m^2^ (after 12 months)	66.1% (±21.6)	Novel CFTR modulators are emerging for the treatment of CF. Specifically, triple combination with ELX/TEZ/IVA has shown to effectively improve both pulmonary and nutritional status in patients with CF with F508del mutation. Body composition should be a part of the routine assessment for patients with CF
Hevilla et al. (2024) [13]	Prospective observational single-center longitudinal study	31	30.7 (±9)	12 months	ETI	+1.21 kg/m^2^ (after 12 months)	70.5% (±21.6)	Despite a reduction in caloric intake, an increase in weight was observed one year after initiating ETI. This increase was attributed to gains in both FM and FFM, suggesting improved metabolic efficiency and nutrient absorption. Both SM and BIA were found to be useful assessment tools. These findings indicate the need to modify the nutritional approach, focusing on the quality rather than the quantity of intake, and aiming for an appropriate body composition (FFM) rather than solely focusing on BMI
Solís-García et al. (2024) [14]	Prospective, observational, longitudinal, multicenter study	108	29.5 ± 9.4	12 months	ETI	+1.1 kg/m^2^	60.3% (±24.2)	CF patients treated with triple therapy experience significant weight gain, increasing the proportion of overweight patients. CF patients who experienced greater weight gain were those with worse BMI at the start of treatment, as well as patients with worse lung function and a greater number of exacerbations in the year before starting ETI therapy
Westhölter et al. (2024) [15]	Retrospective observational study	66	35 years (27.75–42.5), median	Follow-up chest CT scans were performed 148–1147 days after starting ETI, with a median of 529 days	ETI	+1.4 kg/m^2^	70.5% (±20.9)	After receiving ETI therapy, marked increases were observed in all adipose tissue ratios among pwCF, including the total adipose tissue ratio (+46.21%, *p* < 0.001). In contrast, only small, but statistically significant increases in the muscle ratio were measured in the overall study population (+1.63%, *p* = 0.008). Study participants who were initially categorized as underweight experienced more pronounced effects on total adipose tissue ratio (*p* = 0.002), while gains in muscle ratio were equally distributed across BMI categories (*p* = 0.832). Our findings suggest that ETI therapy primarily affects adipose tissues, not muscle tissue, in adults with CF. These effects are primarily observed among pwCF who were initially underweight. Our findings may have implications for future nutritional management of pwCF
Navas-Moreno et al. (2024) [16]	Prospective	26	28.8 years (24.1–35.3), median	6–12 months post-treatment	ETI	+1.20 kg/m^2^	65.6% (±21.6)	CT scans represent an opportunity to assess body composition on CF. Combination treatment with CFTR modulators leads to an improvement in FEV1 and to an increase in body mass in all compartments primarily at the expense of fat mass
Patel et al. (2024) [17]	Retrospective observational study	136	27 years (20–37), median	Post-ETI data were collected > 1 month after starting therapy, with actual measurement windows ranging from 31 to 300 days, median 181 days.	ETI	+1.6 kg/m^2^	66.1% (Total cohort)	After initiation of ETI therapy, serum lipids increased in our population, but most values remained within the normal range. Vitamins A and D levels increased post-ETI and no changes were noted in vitamin E. No significant correlation between the degree of BMI change and the magnitude of increase in lipids or vitamin levels was found
Enaud et al. (2025) [18]	Prospective, real-world, multicenter observational study	62	≈27 years (estimated from children and adult subgroups)	12 months	ETI	+1.20 kg/m^2^	82.3% (±21.0)	ETI therapy enhances nutritional status in pwCF, independently of increased caloric intake. Further research is essential to refine dietary recommendations under ETI treatment, aiming to prevent overweight and obesity while optimizing health outcomes
Loel et al. (2025) [19]	Retrospective cohort study (pre–post ETI comparison)	40	≥18 years	9 ± 3 months	ETI	+1.41 kg/m^2^ (after 12 months)	68.2% (±24.3)	Comparable peripheral muscle mass and function have been demonstrated in pwCF on ETI, albeit a group with good lung function. Research is needed to confirm these findings longitudinally in pwCF, including those with more severe lung disease, who are less physically active, and have less optimal nutrition and exercise support
Clayton et al. (2025) [20]	Cross-sectional, single-center, observational study	30 total (15 pwCF on ETI; 15 age- and sex-matched healthy controls)	24.4 years (14.0–46.4), median	Cross-sectional—no longitudinal follow-up	ETI	+1.4 kg/m^2^	72.1% (±21.0)	BMI was nearly identical between ETI-treated CF patients and healthy controls. pwCF on stable ETI therapy exhibited no differences in peripheral muscle strength, power, endurance, fatigability, or body composition compared with healthy matched controls. Lean mass and muscle strength were strongly correlated in both groups. Findings suggest that in pwCF with good lung function, ETI may normalize muscle mass and function to healthy levels

People with cystic fibrosis (pwCF); body mass index (BMI); forced expiratory volume in 1 s (FEV_1_); normal-weight obesity (NWO); fat mass (FM); free-fat mass (FFM); skeletal mass (SM); bioelectrical impedance analysis (BIA); cystic fibrosis-related diabetes (CFRD). Values are reported as mean ± SD or median (range), as provided in the original studies.

## Data Availability

No new data were created or analyzed in this study. The original contributions presented in this study are included in the article. Further inquiries can be directed to the corresponding author.

## Supplementary Materials

The following supporting information can be downloaded at https://www.mdpi.com/article/10.3390/biology15040367/s1, Table S1: Methodological quality assessment of the included studies.

## Abbreviations

The following abbreviations are used in this manuscript:

BMI	Body Mass Index
CF	Cystic Fibrosis
CFRD	Cystic Fibrosis-Related Diabetes
CFTR	CF Transmembrane Conductance Regulator
CRP	C-Reactive Protein
DXA	Dual-energy X-ray Absorptiometry
ETI	Elexacaftor/Tezacaftor/Ivacaftor
FEV_1_	Forced Expiratory Volume in 1 s
FFM	Fat Free Mass
FM	Fat Mass
HDL/LDL	High/Low-Density Lipoprotein
HR	Hazard Ratio
IGT	Impaired Glucose Tolerance
LUM/IVA	Lumacaftor/Ivacaftor
OGTT	Oral Glucose Tolerance Test
OW/OB	Overweight/Obesity
ppFEV_1_	Percent Predicted Forced Expiratory Volume in 1 Second
VLDMA	Very Low Density Muscle Area
