# Supplementary material for: Beyond BMI: Nutritional Recovery and Functional Implications of CFTR Modulators in Cystic Fibrosis
Source: Biology (Basel). 2026 Feb 22;15(4):367. doi: 10.3390/biology15040367 (PMC12937645; doi:10.3390/biology15040367)
Supplement: Supplementary file 1 [file biology-15-00367-s001.zip › biology-4086924-supplementary.pdf]

Table S1: Methodological quality assessment of the included studies

Author (year)	Selection (0–4)	Comparability (0–2)	Outcome (0–3)	Total /9	Methodological Quality
Enaud et al. (2025) [18]	4	2	3	**9**	High
Loel et al. (2025) [19]	4	2	3	**9**	High
Clayton et al. (2025) [20]	3	1	2	**6**	Moderate
Imrei et al. (2025) [8]	4	2	3	**9**	High
Hevilla et al. (2024) [13]	4	2	3	**9**	High
Solís-García et al. (2024) [14]	4	2	3	**9**	High
Westhölter et al. (2024) [15]	4	2	3	**9**	High
Navas-Moreno et al. (2024) [16]	3	1	2	**6**	Moderate
Patel et al. (2024) [17]	4	2	3	**9**	High
Knott-Torcal et al. (2023) [12]	4	2	3	**9**	High
Taelman et al. (2023) [10]	4	2	3	**9**	High
Tindall et al. (2023) [7]	4	2	3	**9**	High
Caley et al. (2023) [11]	4	2	3	**9**	High
Schembri et al. (2023) [5]	4	2	3	**9**	High
Petersen et al. (2022) [9]	4	2	3	**9**	High
King et al. (2021) [6]	3	1	2	**6**	Moderate
Borowitz et al. (2016) [3]	4	2	3	**9**	High
